# Special Section Guest Editorial: Fluorescence Lifetime Imaging, Optical Micromechanics, and Beyond

**DOI:** 10.1117/1.JBO.25.7.071201

**Published:** 2020-07-22

**Authors:** Stefan Andersson-Engels, Peter E. Andersen

**Affiliations:** a Lund University, Department of Physics, Biophotonics Group, Lund, Sweden; b Tyndall National Institute, IPIC, Biophotonics@Tyndall, Lee Maltings, Cork, Ireland; c University College Cork, Department of Physics, Cork, Ireland; d Technical University of Denmark, Department of Health Technology, Lyngby, Denmark

## Abstract

The editorial introduces the Journal of Biomedical Optics Special Section on Selected Topics in Biophotonics: Fluorescence Lifetime Imaging and Optical Micromechanics.

The present special section of the *Journal of Biomedical Optics* Volume 25 Issue 7, entitled “Fluorescence Lifetime Imaging and Optical Micromechanics,” comprises two invited tutorial papers and several contributed papers from the summer school Biophotonics ’19^1^ as well as contributed papers within the general scope of the school. This section is meant to reflect highlights from the summer school, and serves as an opportunity for participants to publish the work discussed during the school in a collected fashion for everyone to read. After now completing the Biophotonics ’19 summer school with this special section, we are very much looking forward to celebrating the anniversary of the school at Biophotonics ’21 (this school being the 10^th^ in the series^1^) and the accompanying JBO special section.

## Motivation and Purpose of Biophotonics Graduate Schools

Over the past decade, lasers, optical methods, and instruments based on light interaction with tissues have emerged as powerful techniques for medical diagnostics, monitoring wide spectra of tissue function and pathology. In biophysics and biology, optical sensing and manipulation of cells have strengthened understanding of basic cell function. Together with improved laser therapeutic techniques, optical sensing and cell manipulation form the basis for increasing interest in biophotonics. Throughout Europe, the USA, and the rest of the world, major research centers are highly active in this field that in a broad sense may be labeled biophotonics. Therefore, education within this area is increasingly important.

The main purpose with the biennial graduate summer school is to provide education within biophotonics for students and young scientists at the highest international level. Our aim is to attract internationally renowned researchers as lecturers who would attract the most talented young researchers worldwide in the field of biophotonics.

## Format of the Biophotonics Graduate Summer School

The school mainly targets graduate students and postdoctoral fellows from around the world. The format of the school is a combination of lectures and student poster presentations, with time between lectures for discussions and exchange of scientific ideas. The lecturers cover one topic in a full session comprising four lectures, which thoroughly covers the basics and state-of-the-art of each topic. On one hand, this choice limits the number of topics taught at each school. On the other hand, the topics selected for the schools are covered in detail. Therefore, the range of topics taught will change from school to school.

An important feature of the school format is that students and lecturers spend the entire week together, which provides excellent opportunities for the exchange of scientific ideas, networking, and socializing.

The 9^th^ International Graduate Summer School Biophotonics ’19^1^ covered the basics of lasers as well as supercontinuum sources and their application in medicine, tissue optics, photodynamic therapy, optical manipulation and optical tweezers, and their applications in biophotonics, optical micromechanics and speckle (covered in one invited tutorial paper), diffuse optical and molecular imaging, optical coherence tomography, photo-acoustic tomography, fluorescence life-time imaging (covered in another invited tutorial paper), and coherent Raman scattering microscopy. In addition, at the kick-off of the school, entrepreneurship was covered in two keynote lectures. One of these two lectures was given by Dr. Cuccia (Modulim, USA), an alumni of the very first school, Biophotonics 2003, who spoke on his journey from PhD student, to inventor, to CEO of a start-up company selling FDA-approved medical devices based on his own original ideas. Prof. Sune Svanberg (Lund University, Sweden) delivered a third keynote on spectroscopy in life sciences.

## Special Section in the *Journal of Biomedical Optics*

We are pleased to introduce the contributions to this special section on “Fluorescence Lifetime Imaging and Optical Micromechanics” comprising two invited tutorial papers and four contributed papers, from participants of the school and from other researchers in the field. Not all of the school-participant contributions fall within the themes covered by the special section, but all contributions reflect the core topics of the school and span the fields of biomedical optics and biophotonics. The invited tutorial papers are:

• Rupsa Datta et al., “Fluorescence lifetime imaging microscopy: fundamentals and advances in instrumentation, analysis, and applications,” *J. Biomed. Opt.* **25**, 071203 (13 May 2020), doi 10.1117/1.JBO.25.7.071203
• Zeinab Hajjarian and Seemantini K. Nadkarni, “Tutorial on laser speckle rheology: technology, applications, and opportunities,” *J. Biomed. Opt*. **25**, 050801 (1 May 2020), doi 10.1117/1.JBO.25.5.050801.

The first paper, from a lecturer (Prof. Melissa Skala) at the school, provides an excellent background to the field of fluorescence lifetime imaging microscopy, whilst also reviewing instrumentation and applications. The second invited tutorial, also from a lecturer (Prof. Seemantini Nadkarni) at the school, covers speckle-based measurement techniques and their applications for imaging cell micromechanics. These tutorials further build the online JBO resources for high quality teaching material and provide a natural continuation to previous tutorial papers on the foundation of diffuse optics,^2^ imaging thick tissues with diffuse optics,^3^ molecular imaging,^4^ optical micromanipulation,^5^ photodynamic therapy,^6^ optical coherence tomography,^7^ biological imaging with coherent Raman scattering microscopy,^8^ *in vivo* applications of Raman spectroscopy,^9^ photoacoustic tomography,^10^ and fiber-based sources for biophotonics^11^ published in similar special sections from previous schools. These papers all belong to a planned series of tutorial review papers from each biennial school that provide **high-level,** ***open-access*** **educational material for the benefit of the scientific community** and, in addition, fulfill our own motivation for creating the school in the first place. With this special section, there are now in total 12 excellent tutorials that provide profound introduction into the basics of our field.

In addition to the invited tutorial papers, we have four contributed papers. Erkkiläet al.10.1117/1.JBO.25.7.071202 demonstrate a surgical microscope platform with fluorescence lifetime imaging for neurosurgery. They demonstrate the capabilities in guiding of surgery for 5-ALA-labeled glioblastoma tissue. Jameset al.10.1117/1.JBO.25.7.071204 demonstrate fluorescence lifetime imaging in a two-photon microscope, i.e., combining tomography and lifetime measurements, investigating sentinel lymph nodes. Calcium-induced epidermal differences are investigated *in vitro* by using two-photon microscopy to better understand its diagnostic potential (label-free biomarker) by Malaket al.10.1117/1.JBO.25.7.071205 Finally, Meyeret al.10.1117/1.JBO.25.7.071206 present a method for optimal selection of emission spectral bands in multiphoton microscopy of biomarkers. The technique is demonstrated using autofluorescence spectra of 2D and 3D cell cultures.

## References

[1] “International Graduate Summer School: Biophotonics,” www.biop.dk. 10.1117/1.JBO.27.5.050102 PMC9124642 35599336

[2] Jacques S. L. Pogue B. W., “Tutorial on diffuse light transport,” J. Biomed. Opt. 13, 041302 (2008). JBOPFO 1083-3668 10.1117/1.2967535 19021310

[3] O’Sullivan T. D. et al., “Diffuse optical imaging using spatially and temporally modulated light,” J. Biomed. Opt. 17, 071311 (2012). JBOPFO 1083-3668 10.1117/1.JBO.17.7.071311 22894472 PMC3607494

[4] Sevick-Muraca E. M. Rasmussen J. C., “Molecular imaging with optics: primer and case for near-infrared fluorescence techniques in personalized medicine,” J. Biomed. Opt. 13, 041303 (2008). JBOPFO 1083-3668 10.1117/1.2953185 19021311 PMC2915929

[5] Stevenson D. J. Gunn-Moore F. Dholakia K., “Light forces the pace: optical manipulation for biophotonics,” J. Biomed. Opt. 15, 041503 (2010). JBOPFO 1083-3668 10.1117/1.3475958 20799781

[6] Svanberg K. et al., “Photodynamic therapy: superficial and interstitial illumination,” J. Biomed. Opt. 15, 041502 (2010). JBOPFO 1083-3668 10.1117/1.3466579 20799780

[7] Drexler W. et al., “Optical coherence tomography today: speed, contrast, and multimodality,” J. Biomed. Opt. 19, 071412 (2014). JBOPFO 1083-3668 10.1117/1.JBO.19.7.071412 25079820

[8] Alfonso-García A. et al., “Biological imaging with coherent Raman scattering microscopy: a tutorial,” J. Biomed. Opt. 19, 071407 (2014). JBOPFO 1083-3668 10.1117/1.JBO.19.7.071407 PMC4019423 24615671

[9] Cordero E. et al., “In vivo Raman spectroscopy: from basics to applications,” J. Biomed. Opt. 23, 071210 (2018). JBOPFO 1083-3668 10.1117/1.JBO.23.7.071210 29956506

[10] Lin L. et al., “In vivo photoacoustic tomography of myoglobin oxygen saturation,” J. Biomed. Opt. 21, 061002 (2016). JBOPFO 1083-3668 10.1117/1.JBO.21.6.061002 PMC5397140 26719943

[11] Taylor J. R., “Tutorial on fiber-based sources for biophotonic applications,” J. Biomed. Opt. 21, 061010 (2016). JBOPFO 1083-3668 10.1117/1.JBO.21.6.061010 27286187

